# Contamination of Human Bocavirus Genotypes 1, 2, 3, and 4 in Environmental Waters in Thailand

**DOI:** 10.1128/spectrum.02178-21

**Published:** 2021-12-22

**Authors:** Kattareeya Kumthip, Pattara Khamrin, Arpaporn Yodmeeklin, Hiroshi Ushijima, Niwat Maneekarn

**Affiliations:** a Department of Microbiology, Faculty of Medicine, Chiang Mai University, Chiang Mai, Thailand; b Center of Excellence (Emerging and Re-emerging Diarrheal Viruses), Chiang Mai University, Chiang Mai, Thailand; c Department of Developmental Medical Sciences, The University of Tokyo, School of International Health, Graduate School of Medicine, Tokyo, Japan; d Department of Pathology and Microbiology, Nihon University School of Medicine, Tokyo, Japan; Wright State University

**Keywords:** human bocavirus, environmental water, genotype, waterborne transmission, Thailand

## Abstract

Human bocavirus (HBoV) has been recognized as one of the common pathogens which cause respiratory disease and acute gastroenteritis in children worldwide. Recently, our studies reported the detection of HBoV in children with acute gastroenteritis and in oysters in Thailand. However, studies on the presence of HBoV in environmental waters in Thailand have not yet been conducted. In this study, 126 environmental water samples collected from November 2016 to July 2018 were investigated. Detection of HBoV was based on amplification of the VP1/VP2 region of the HBoV genome by nested PCR followed by nucleotide sequencing and phylogenetic analysis. HBoV was detected in 34 out of 126 samples (27.0%). All four HBoV genotypes, HBoV1 to HBoV4, were detected. HBoV2 was the most frequently detected genotype (61.8%), followed by HBoV1 (23.5%), HBoV4 (8.8%), and HBoV3 (5.9%). The highest detection rate of HBoV was observed during the warmest months in Thailand: April 2017 and March 2018. Phylogenetic analysis of VP1/VP2 nucleotide sequences of HBoV genotypes revealed that all four of the genotypes detected in environmental waters were closely related to genotypes detected in patients with acute gastroenteritis, which had been reported previously in the same geographical area. This study reports the existence of multiple HBoV genotypes in environmental waters and provides evidence of a considerably high magnitude of HBoV contamination in these waters. These findings demonstrate the potential risk of waterborne transmission of HBoV to humans.

**IMPORTANCE** Recently, we reported the detection of HBoV genotypes 1, 2, and 3 in pediatric patients with acute gastroenteritis, and the detection of HBoV1 and 2 in oysters in Thailand. In this study, we reported the detection of HBoV1, 2, 3, and 4 contamination in environmental waters within the same geographic area. Phylogenetic analysis demonstrated that the HBoV genotypes detected in environmental waters and in oysters were closely related to HBoV detected in patients. These findings imply that HBoV contamination in oysters and in environmental waters could be a potential sources of foodborne and waterborne transmission to humans.

## INTRODUCTION

Human bocavirus (HBoV) is a small nonenveloped virus with an icosahedral capsid, approximately 25 nm in size. HBoV belongs to the family *Parvoviridae*, in the genus *Parvovirus*. Its genome is a linear single-stranded DNA about 5.3 kb in length, with 3 open reading frames (ORF1, ORF2, and ORF3) encoding nonstructural (NS1 and NP1) and structural (VP1 and VP2) proteins (1). HBoV is classified into 4 genotypes, HBoV1 to HBoV4. HBoV2 is further subdivided into 2 subtypes, 2A and 2B. HBoV has been recognized globally as a common pathogen found in children with respiratory infection and gastroenteritis. HBoV1 is usually detected in children with respiratory tract infections (2–4), while HBoV2, HBoV3, and HBoV4 are generally detected in stool samples from children with acute gastroenteritis (5–7). HBoV is most likely transmitted by fecal-oral and respiratory routes. The global prevalence of HBoV infection in patients with respiratory disease and acute gastroenteritis has been reported at rates of 1.0% to 56.8% and 1.3% to 63%, respectively (8). In recent years, the presence of HBoV in several types of environmental samples, including river water, wastewater, sewage, and surface water, has been documented with prevalence ranging from 3.7% to 81% (9–13). Moreover, HBoV contamination in food materials, particularly in shellfish, has been recently reported (14–16). The cocirculation of HBoV in humans, environmental waters, and oysters suggests a potential risk of foodborne and waterborne transmission to humans, as well as indicating that water and shellfish might be important vehicles for HBoV transmission to humans. Although a number of studies performed in this region have reported HBoV occurrences in clinical specimens from patients with respiratory and gastrointestinal infections, the presence of HBoV in environmental waters in Thailand is largely unknown. Recently, our group reported the prevalence of HBoV in patients with acute gastroenteritis (17) and demonstrated HBoV contamination in oyster samples (14). It is interesting to investigate the presence of HBoV in environmental waters and to analyze the genetic relationship among HBoV strains circulating in humans, environmental waters, and food materials. To fill the gap and address potential sources of HBoV contamination, this study investigated the occurrence and genotype diversity of HBoV circulating in various types of environmental waters in Thailand.

## RESULTS

### Detection of HBoV in environmental water samples.

A total of 126 environmental water samples, collected from six different locations in Chiang Mai, Thailand between November 2016 and July 2018, were tested for the presence of HBoV. In this study, the prevalence of HBoV was 27.0% (34 out of 126) (Table 1). It was found that all six sampling locations were contaminated with HBoV, with prevalence varying from 9.5% to 66.7%. In addition, all four HBoV genotypes (HBoV1 to HBoV4) were detected in this study. HBoV2 was the most predominant genotype (61.8%) followed by HBoV1 (23.5%), HBoV4 (8.8%), and HBoV3 (5.9%). Among the sampling sites, high frequencies of HBoV contamination were observed in the Mae Kha Canal (wastewater; 66.7%) and the Suandok Canal (irrigation water; 42.9%). Considering genotype diversity of HBoV detected in different sampling sites, it was found that three genotypes (HBoV2, HBoV3, and HBoV4) were detected in wastewater (Mae Kha Canal). In irrigation water (Suandok and Sompech canals), two or three HBoV genotypes (HBoV1, HBoV2, and HBoV4) were detected. HBoV1 and HBoV2 were detected in river water (Ping River). In environmental reservoirs, only HBoV1 was detected in the Ang Kaew Reservoir, while HBoV1, HBoV2, and HBoV3 were found in the Buak Hard Garden (Table 1). It should be noted that the Mae Kha (wastewater) and Suandok canal (irrigation water) were the first and second most polluted sites, respectively: these two sites were contaminated with three HBoV genotypes, while the other sites were contaminated with only one or two HBoV genotypes.

**TABLE 1:** Detection of HBoV genotypes in environmental waters in Chiang Mai, Thailand

Sampling location	No. of samples tested	No. of positive samples (%)	HBoV genotype (%)			
			HBoV1	HBoV2	HBoV3	HBoV4
Ang Kaew Reservoir^*a*^	21	2 (9.5)	2	0	0	0
Buak Hard Garden^*a*^	21	4 (19.0)	2	1	1	0
Suandok Canal^*b*^	21	9 (42.9)	2	6	0	1
Sompech Canal^*b*^	21	2 (9.5)	1	1	0	0
Ping River^*c*^	21	3 (14.3)	1	2	0	0
Mae Kha Canal^*d*^	21	14 (66.7)	0	11	1	2
Total	126	34 (27.0)	8(23.5)	21(61.8)	2(5.9)	3(8.8)

a Environmental reservoir.

b Irrigation canal.

c River water.

d Wastewater.

### Seasonality and diversity of HBoV contamination.

**FIG 1:**
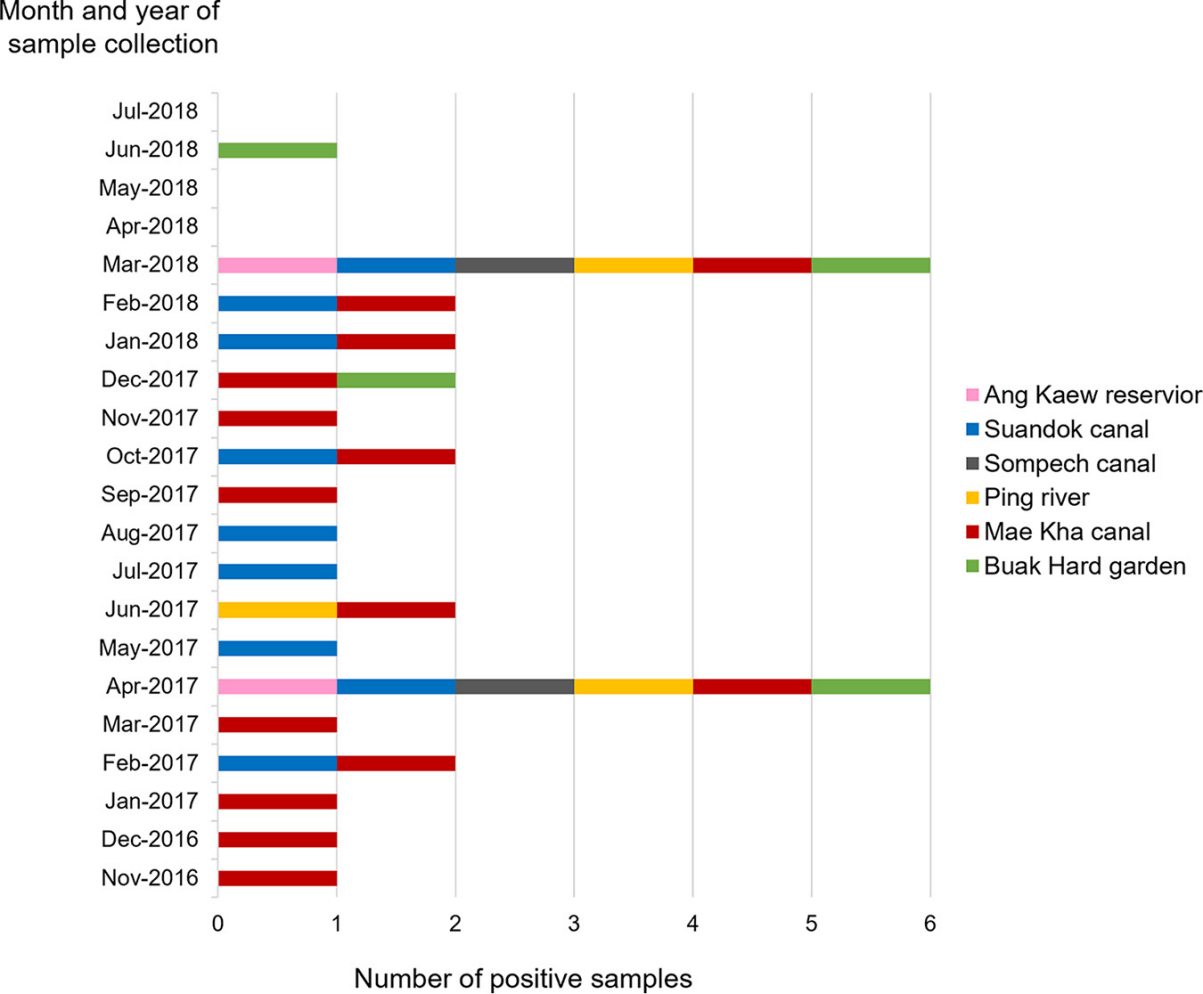
Distribution of HBoV detection in different locations in Chiang Mai, Thailand from November 2016 to July 2018.

**FIG 2:**
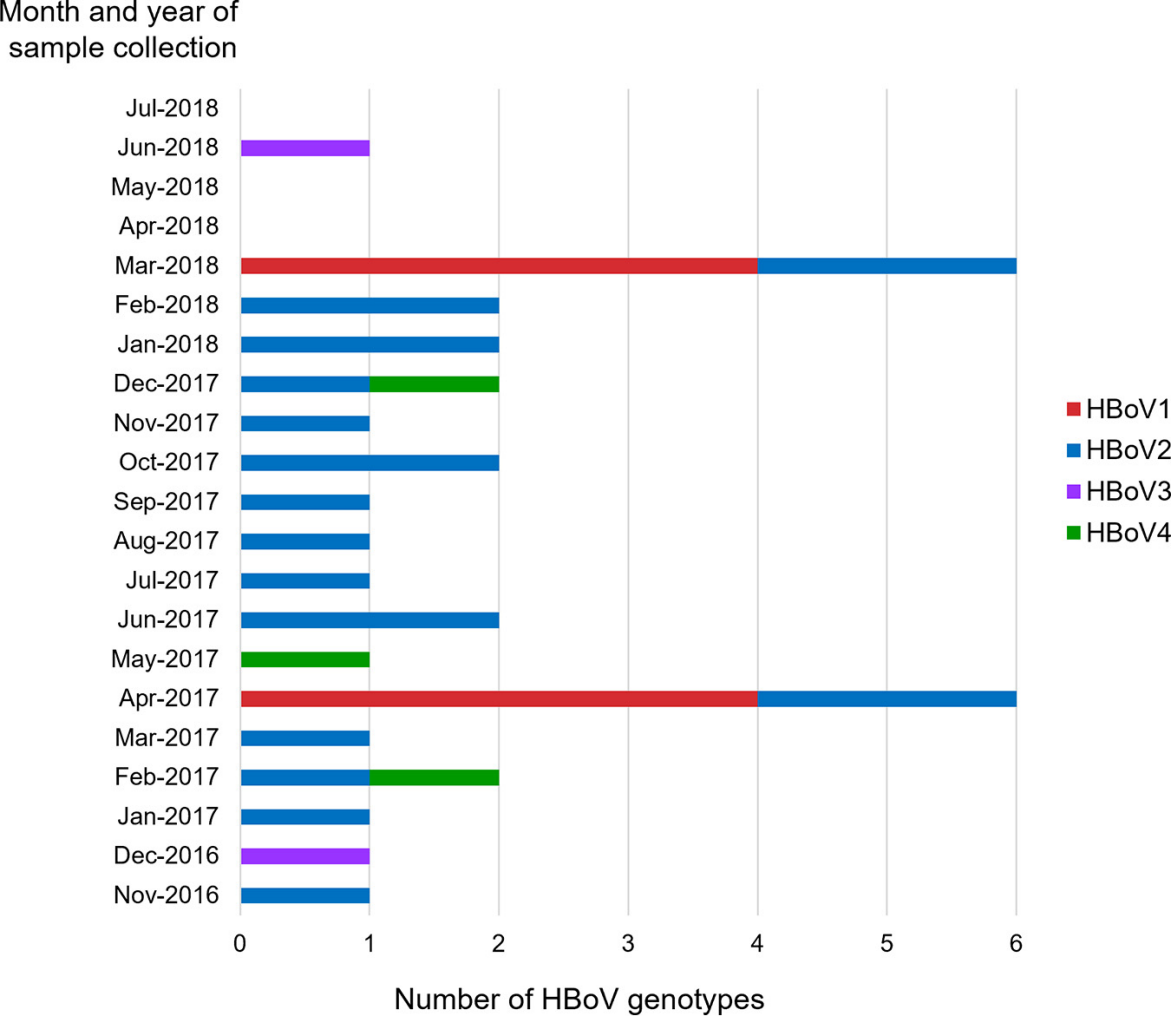
Monthly distribution of HBoV genotypes in environmental water samples from November 2016 to July 2018.

HBoV was continually detected during each month from November 2016 to March 2018 (Fig. 1). From April to July of 2018, HBoV was detected only in June. The peak HBoV detection rate occurred during the summer season in Thailand (i.e., April of 2017 and March of 2018) as all samples collected during these months were 100% positive for HBoV. Two predominant HBoV genotypes, HBoV1 and HBoV2, were detected during these months (Fig. 2). The most predominant genotype, HBoV2, was commonly detected throughout the study period, while HBoV1 was detected only in April 2017 and March 2018. HBoV3 was detected in December 2016 and June 2018, whereas HBoV4 was found in February, May, and December of 2017, but not in 2018.

### Phylogenetic analysis of HBoV genotypes detected in environmental water samples.

**FIG 3:**
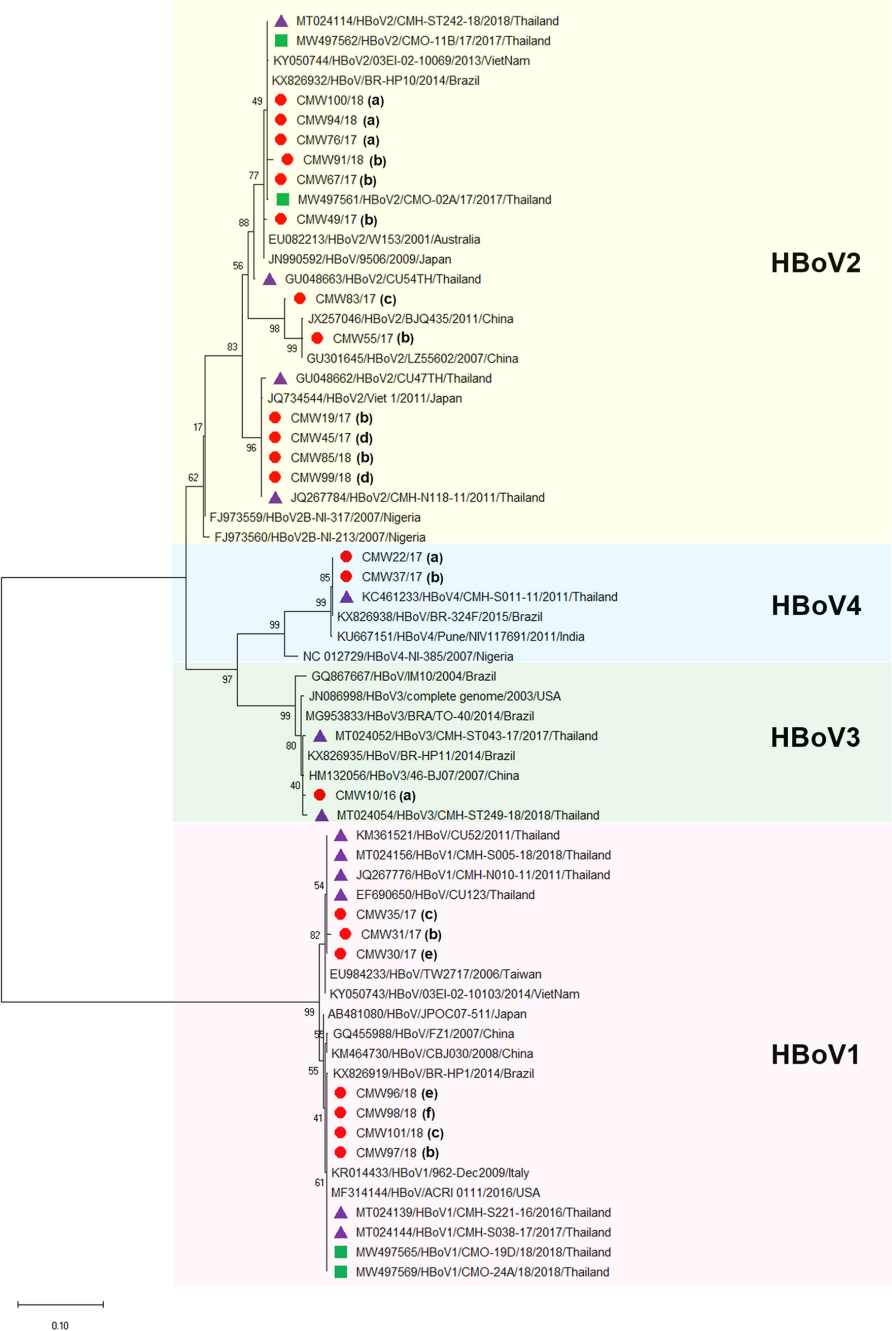
Phylogenetic tree of partial VP1/VP2 genes of HBoV1 to HBoV4 genotypes detected in environmental waters in Chiang Mai, Thailand. The HBoV strains detected in this study are indicated by a red symbol. Sampling locations Mae Kha Canal, Suandok Canal, Buak Hard Garden, Ping River, Ang Kaew Reservoir, and Sompech Canal are marked by (a), (b), (c), (d), (e), and (f), respectively. HBoV strains detected in patients with acute gastroenteritis and in oyster samples in Thailand are indicated by purple and green symbols, respectively. The scale bar indicates the number of nucleotide substitutions per site and a bootstrap value of 1,000 replicates was used.

Screening for HBoV in environmental waters revealed that, of the 34 HBoV strains detected in this study, 8, 21, 2, and 3 were HBoV1, HBoV2, HBoV3, and HBoV4, respectively. However, only 22 out of 34 HBoV strains yielded high-quality sequencing results for phylogenetic analysis. Thus, representatives of these four genotypes, including 7, 12, 1, and 2 strains of HBoV1, HBoV2, HBoV3, and HBoV4, respectively, were further analyzed by phylogenetic analysis. The results showed that the HBoV1 strains detected in this study were closely related to HBoV1 strains reported previously in human stool samples, respiratory samples, and oysters in Thailand, sharing 98.0% to 100% nucleotide sequence homology; the strains in this study were also related to HBoV1 strains detected in clinical samples in other countries around the world, including Taiwan, Vietnam, Japan, China, Brazil, Italy, and the United States, sharing 97.0% to 100% nucleotide sequence identity (Fig. 3). The HBoV2 strains detected in this study were similar to HBoV2 strains reported in human stool samples from Thailand, Vietnam, Japan, China, and Brazil, sharing 93.2% to 100% nucleotide sequence homology to HBoV strains reported previously in Thailand and 90.9% to 100% homology to strains reported previously in other countries. The HBoV3 strains detected in this study were most closely related to HBoV3 strains reported previously in human stool samples from Thailand (99.0% to 100% nucleotide sequence identity) while the HBoV4 strains had 100% nucleotide sequence identity to HBoV4 strains reported previously in human stool samples from Thailand.

## DISCUSSION

Recently, several studies have reported the dissemination of HBoV into the environment. The presence of HBoV in various types of environmental waters, including river water, wastewater, and sewages, has been documented with frequencies ranging from 37.5% to 100% (9–13). In this study, HBoV was detected in several types of environmental waters in Thailand with an overall frequency of 27.0%, slightly lower than that reported in other studies. However, when looking only at wastewater samples, the HBoV detection rate is 66.7% (Table 1), which is in good agreement with reports from other countries where detection rates were reported at 38% to 100% in sewage/wastewater (9, 10, 12, 13, 16) and 37.5% to 40.8% in river water (11, 18).

HBoV infection is commonly associated with respiratory and gastroenteritis diseases worldwide (8). HBoV1 infection mainly involves respiratory tract infections (2–4, 19) while HBoV2, HBoV3, and HBoV4 are associated with gastrointestinal tract infections as they are usually detected in stool samples (5–7, 20, 21). Regarding HBoV contamination in the environment, HBoV2 and HBoV3 are significantly more prevalent in environmental water than HBoV1, whereas HBoV4 is rarely detected (9, 10, 12, 13, 16, 18, 22). In this study, HBoV2 (61.8%) and HBoV1 (23.5%) were more prevalent than HBoV3 (5.9%) and HBoV4 (8.8%). This predominance of HBoV2 in environmental waters is in agreement with reports from other countries worldwide. Nevertheless, in this study, HBoV1 was detected at a much higher rate than that of the HBoV3 genotype. The variation in detection rate of HBoV genotypes in environmental waters may be linked to the HBoV genotypes circulating in humans in the same geographical area. In fact, HBoV1 and HBoV2 have been reported as the most predominant genotypes circulating in children with acute gastroenteritis in Chiang Mai, Thailand (6, 17), where HBoV2 and HBoV1 have been demonstrated, in this study, as the first and second most predominant genotypes, respectively.

Phylogenetic analysis was performed on 22 representatives out of the 34 HBoV strains detected in this study. Although we were able to amplify and sequence all positive samples, the sequencing results of some samples were shortened and could not be analyzed together with the other samples. However, genotyping based on the basic local alignment search tool (BLAST) tool could identify the genotypes of all HBoV strains detected in this study. The phylogenetic tree revealed that the HBoV strains detected in the current study were closely related to HBoV strains detected in patients with acute gastroenteritis from several countries in Asia (Thailand, China, Japan, Vietnam, Taiwan, and India) and from other countries outside Asia (Australia, Brazil, Italy, and the United States), sharing high nucleotide sequence homology (90.9% to 100%). Interestingly, the HBoV strains detected in this study shared very high nucleotide sequence identities (98.0% to 100%) with HBoV strains detected previously in human stool samples and oyster samples in the same geographical area, indicating an epidemiological link between infectious HBoV strains and those contaminating environments. Furthermore, patients with acute gastroenteritis associated with HBoV-contaminated drinking water have been documented in Finland (23), suggesting potential waterborne transmission of HBoV. Taken together, the results of this study suggest that environmental waters could be a potential source of the virus and play an important role in its transmission to humans. In addition, our findings may suggest that the diversity of HBoV genotypes with high prevalence rates in environmental waters is reflective of HBoV circulation in the community; our data collected from wastewater showed the highest rate of bocavirus DNA among the four water sample types. However, more information about human activities/industries (e.g., oyster farming, fishing) conducted in the vicinities of the 6 sampling locations should be obtained to justify the actual risk of HBoV infection via environmental waters.

In conclusion, this report investigates HBoV contamination in environmental waters in Thailand for the first time and suggests the potential risk of waterborne transmission of HBoV to humans.

## MATERIALS AND METHODS

### Sample collection and concentration.

Environmental water samples were collected from six different locations in Chiang Mai, Thailand from November 2016 to July 2018. One-hundred-mL samples of environmental water were collected from each location once a month throughout the study period. These sampling locations included various types of water such as an environmental reservoir (Ang Kaew Reservoir and Buak Hard Garden), irrigation water (Suandok Canal and Sompech Canal), river water (Ping River), and wastewater (Mae Kha Canal). Virus particles in the environmental water samples were concentrated using the polyethylene glycol (PEG) precipitation method as described previously (24). Briefly, 8 g of PEG 6000 and 2.3 g of NaCl were added into 100-mL water samples and stirred at 4°C overnight. Samples were then centrifuged at 10,000 × *g* for 30 min at 4°C. After centrifugation, the supernatant was discarded, and the pellet was resuspended in 200 μL RNase-free water. Then, 200 μL of concentrated sample was extracted for viral nucleic acid using Geneaid Viral Nucleic Acid Extraction Kit (Geneaid, Taiwan) according to the manufacturer’s instruction. The obtained final volume of 50 μL nucleic acid extract was used for further steps.

### Detection of human bocavirus.

To detect the presence of HBoV in the samples, nested PCR targeting the VP1/VP2 region was performed using GoTaq DNA Polymerase (Promega, USA) together with the first-round primers AK-VP-F1 and AK-VP-R1 and the second-round primers AK-VP-F2 and AK-VP-R2, as described previously (25). For the first-round PCR, 2.0 μL of DNA template was added into 23 μL of PCR mixture containing first-round primers (0.4 μM each), nuclease-free water, and GoTaq Green Master Mix. The thermocycling conditions for the first-round PCR were as follows: 94°C for 3 min followed by 35 cycles of 94°C for 1 min, 55°C for 1 min, and 72°C for 1 min, with a final extension at 72°C for 10 min. For the second-round PCR, 2.0 μL of the first-round PCR product was used as the template and the annealing step was done at 58°C. The first- and second-round PCR generated amplicon sizes of 609 bp and 576 bp, respectively.

Throughout the process of viral genome extraction and nested PCR, the known HBoV-positive and -negative stool samples were included as positive and negative controls, respectively. In addition, the detection limit of HBoV by nested PCR was determined by making 10× serial dilutions of extracted DNAs with concentrations of 1.5 × 10^0^ to 1.5 × 10^−6 ^ng/μL and testing them by nested PCR. The detection limit of HBoV by nested PCR in this study was 1.5 × 10^−2 ^ng/μL.

### Nucleotide sequencing and phylogenetic analysis.

The PCR products were purified using a Gel/PCR DNA Fragment Extraction Kit (Geneaid, Taiwan) according to the manufacturer’s protocol. After purification, the purified PCR product DNA was checked for DNA quality by agarose gel electrophoresis and quantified using a NanoDrop Spectrophotometer. A sufficient and optimal concentration of purified PCR product (20 ng/μL) with AK-VP-F2 primer was used for direct nucleotide sequencing, which was provided by First BASE Laboratories (First BASE Laboratories, Malaysia). After Sanger sequencing, nucleotide sequences of HBoV strains detected in this study were compared with those of reference sequences retrieved from the NCBI GenBank database, using the BLAST (https://blast.ncbi.nlm.nih.gov/Blast.cgi). The reference sequences which had high nucleotide sequence identity to HBoV strains detected in this study (as analyzed by BLAST) and representative sequences of different HBoV genotypes from humans and oysters collected during the same period in Thailand were included in the phylogenetic analysis. To construct a phylogenetic tree of partial VP1/VP2 regions, MEGA X software (26) was used. Evolutionary history was generalized using the Maximum Likelihood method with the best-fit model, Hasegawa-Kishino-Yano, (27) complemented by the MEGA X software (26).

### Data availability.

Sequences of HBoV strains detected in this study were deposited in the GenBank database under the accession numbers OK244663 to OK244684.
